# Thermal and oxidative stress responses of *Paecilomyces* species recovered from beverage processing environments

**DOI:** 10.1007/s11274-026-04970-6

**Published:** 2026-04-27

**Authors:** Mailah Ali Abdul Rahman Mahfouz, Arthur Kael Rodrigues Pia, Rafaella Santos Mori Bragil, Naara Aparecida Almeida, Anderson S. Sant’Ana, Benedito Corrêa, Rodrigo Savio Pessoa, Liliana de Oliveira Rocha

**Affiliations:** 1 Department of Food Science and Nutrition (DECAN), Faculty of Food Engineering (FEA), State University of Campinas (UNICAMP), Rua Monteiro Lobato, 80, Campinas, São Paulo, 13083-862 Brazil; 2 Department of Microbiology (ICB), Institute of Food Bioscience, University of São Paulo, São Paulo, Brazil; 3 Plasmas and Processes Laboratory, Technological Institute of Aeronautics (ITA), São José dos Campos, São Paulo, Brazil

**Keywords:** Fungal adaptation, Cold plasma, Thermal resistance, Juice spoilage, Physiological response

## Abstract

*Paecilomyces* is a frequently genus associated with food spoilage worldwide. Among its species, *P. variotii* produces the most heat-resistant conidia described to date, while *P. niveus* and *P. dactylethromorphus* are potential patulin producers. Recently, *P. paravariotii* was described as a closely related species to *P. variotii* and the occurrence in food-related environments remains scarcely reported. In this study *Paecilomyces* species associated with orange juice processing environments and spoiled products were characterized using multilocus phylogenetic analysis, and strain-level variability in stress tolerance was evaluated under thermal and non-thermal conditions. Twelve isolates were identified and following preliminary heat screening at 58 and 59 °C, six representative strains were selected for thermal inactivation kinetics at 60 °C and exposure to direct cold plasma generated by a gliding arc plasma jet (GAPJ). Phylogenetic analysis revealed a predominance of *P. paravariotii*, along with *P. variotii*, *P. dactylethromorphus*, and *P. lecythidis*. Thermal assays showed wide variability, with *P. variotii* being the most resistant and *P. dactylethromorphus* the most sensitive. Responses to GAPJ-induced oxidative stress mirrored the thermal resistance profiles observed among the strains, with the most resistant strains remaining viable after a 20 to 30-minute exposure. Overall, strain-level variability reflects ecological and environmental influences on fungal stress adaptation, emphasizing the importance of strain-level analyses and ecological context in understanding fungal persistence in food systems.

**Graphical Abstract:**

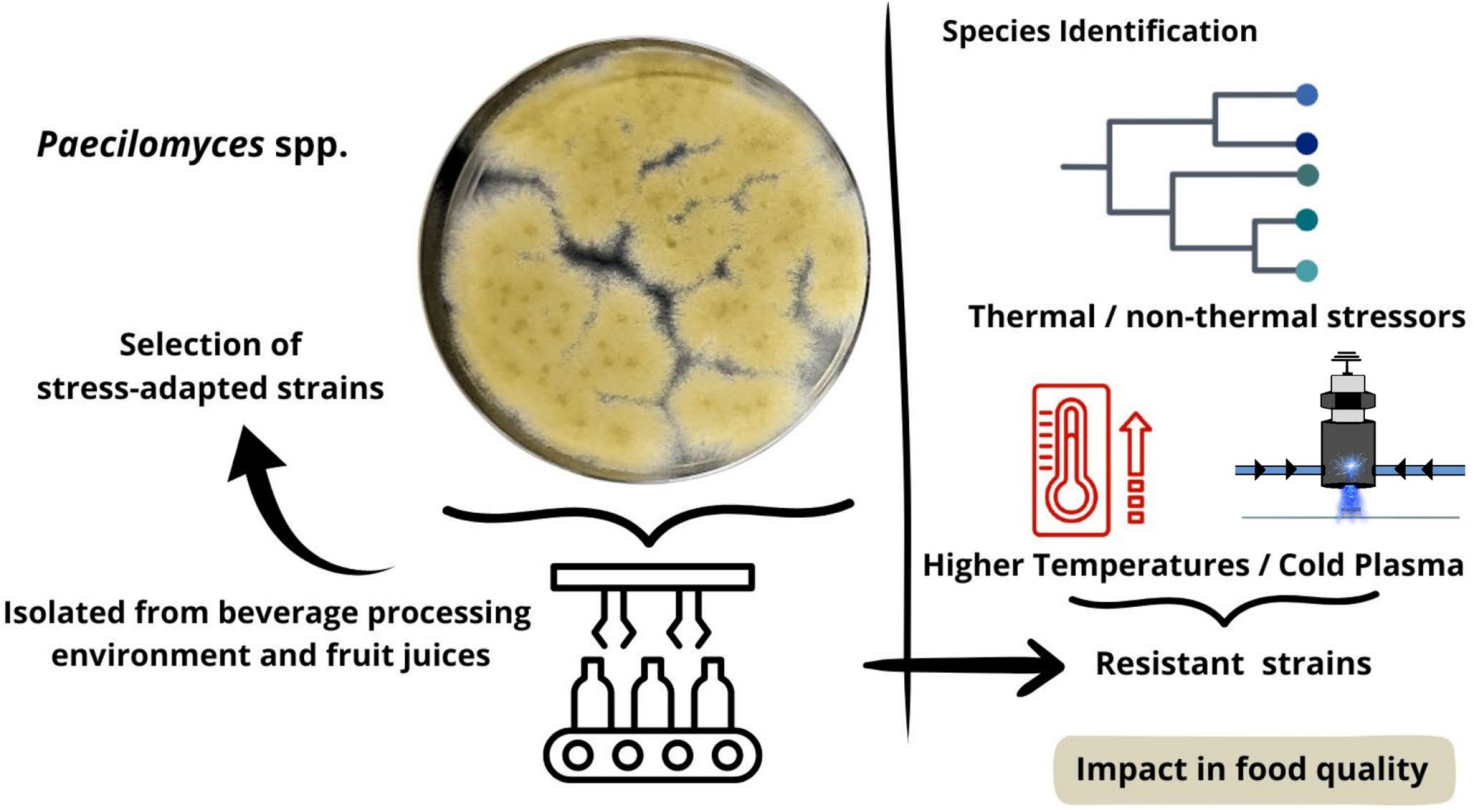

**Supplementary Information:**

The online version contains supplementary material available at 10.1007/s11274-026-04970-6.

## Introduction

Fungi present significant challenges to the food chain, primarily due to their ability to spoil various food matrices. In addition, certain species are able to produce mycotoxins, toxic secondary metabolites that may cause acute or chronic intoxication in vertebrate animals, thereby, compromising food quality and safety (Jafarzadeh et al. 2023; Peloso et al. 2024; Pitt and Hocking 2022).

Raw materials are considered one of the primary sources of fungal contamination in industrial processes. Fungal conidia are easily dispersed through the air and can settle on surfaces and equipment, particularly in environments with inadequate hygiene and sanitization practices (Bernardi et al. 2019). From a physiological perspective, some strains may withstand various stress conditions, including nutrient limitation, wide pH ranges, low water activity (a_w_), and reduced oxygen availability. Moreover, certain fungal species can resist commonly used food preservatives and, in some cases, even survive thermal treatments such as pasteurization, allowing them to persist into the food processing niche (Carvalho et al. 2024).

In this respect, *Paecilomyces* emerges as a particularly important genus due to its ubiquity and its frequent occurrence in heat-treated, acidic, and low-a_w_ food products (Hosoya et al. 2014; Snyder 2022). Several species, including the homothallic *P. fulvus* and *P. niveus*, produce ascospores with high thermal resistance, showing D-values of several minutes at 80–85 °C (Dijksterhuis et al. 2018). The ascospores of the heterothallic species *P. variotii* can survive up to an hour at 85 °C (Houbraken et al. 2005). Moreover, the conidia of *P. variotii* are particularly heat-resistant, with some strains surviving exposure to 60 °C for up to 22 min, depending on the strain (Hosoya et al. 2014; van den Brule et al. 2020a; Brule et al. 2020b). Of particular concern is the toxigenic potential of several *Paecilomyces* species, including patulin production by *P. niveus* and *P. dactylethromorphus*, as well as the synthesis of other toxic secondary metabolites such as byssochlamic acid and viriditoxin by related taxa (Frisvad 2018; Houbraken et al. 2005; Visagie et al. 2024).

The taxonomy of *Paecilomyces* spp. has historically been defined by the morphology asexual and sexual structures. The paucity and variability of these characters led to a broad definition of the genus that was later demonstrated to be a polyphyletic group across two subclasses, *Eurotiomycetidae* and *Sordariomycetidae* (Luangsa-Ard et al. 2004). With the advent of multiloci phylogenetic analysis, some species were relocated to different genera, such as *Purpureocillium lilacinum* and *Phialemonium inflatum*; previously identified as *Paecilomyces lilacinus* and *P. inflatus*, respectively (Luangsa-Ard et al. 2011; Perdomo et al.,^2013^). Currently, the genus comprises 16 accepted species: *P. brunneolus*,* P. clematidis*,* P. dactylethromorphus*,* P. divaricatus*,* P. formosus*,* P. fulvus*,* P. lagunculariae*,* P. lecythidis*,* P. lignorum*,* P. maximus*,* P. niveus*,* P. paravariotii*,* P. penicilliformis*,* P. tabacinus*,* P. variotii* and *P. zollerniae*. The recent inclusion of *Paecilomyces paravariotii*, described as a cryptic sister species of *P. variotii*, and *P. lignorum* reflects ongoing taxonomic refinement within the genus (Urquhart and Idnurm 2023; Visagie et al. 2024, 2025).

*Paecilomyces* species exhibit high ecological versatility and have been isolated from a wide range of environments, including decaying plant material, marine sediments, insects, nematodes, plant rhizospheres, and even areas heavily contaminated with cesium-137 (Lim et al. 2021; Moreno-Gavíra et al. 2020). In food systems, representative species are frequently detected across diverse matrices, ranging from raw commodities (e.g., grains, oilseed, fruits and vegetables) to processed foods such as beverages, butter, pasta, and other heat-treated products. Beyond their ability to degrade common food preservatives, including ascorbic and benzoic acids, these fungi exhibit pronounced adaptability to multiple environmental stressors, enabling their persistence under adverse processing and storage conditions (Moreira et al. 2018).

Given the widespread distribution of *Paecilomyces* species, their resistance to heat and chemical stressors, and their involvement in food spoilage and mycotoxin production, accurate species identification is important for understanding strain-level variability in stress tolerance and ecological adaptation and, therefore, aiding in food safety protocols (Davies et al. 2021; Monpierre et al. 2022; Moreira et al. 2018). Conventional decontamination methods, such as pasteurization and chemical preservatives, may not fully eliminate some strains, favoring the persistence of stress-tolerant strains due to selection pressure of the environment (Dabija et al. 2025).

In this context, increasing attention has been directed toward non-thermal physical stressors as tools to investigate fungal tolerance to extreme environmental conditions. These include ionizing and non-ionizing radiation, pulsed electric fields, pulsed light, ultrasound, high-pressure processing, and, more recently, cold plasma. Rather than functioning solely as preservation technologies, such stressors provide controlled models to evaluate microbial responses to oxidative, electrical, and radiation-associated challenges that differ fundamentally from thermal stress (Lemos et al. 2025; Mirza Alizadeh et al. 2021).

Cold plasma is generated from the ionization of a gas by supplying energy in the form of an electric field, transforming it into the fourth state of matter (Patil et al. 2024). It can be thermal when formed at high temperatures and pressure or non-thermal when produced with less energy (Desai et al. 2024; Kaur et al. 2024). Several discharge configurations have been described, including dielectric barrier discharge, corona discharge, radio frequency, microwave, and gliding arc systems, each generating distinct physicochemical stress environments depending on reactor geometry, gas composition, and operating parameters (Cherif et al. 2023; Lemos et al. 2025).

The biological effects of cold plasma exposure are primarily attributed to the combined action of reactive oxygen and nitrogen species (ROS and RNS, respectively), and with UV radiation. In filamentous fungi, the effects of this technology involve interactions of reactive species with the cell wall and membrane, reduction of ergosterol content, and degradation of proteins and nucleic acids; which can lead to apoptosis (Desai et al. 2024; Meneses-Espinosa et al. 2024; Neuenfeldt et al. 2023). From a stress-biology perspective, cold plasma exposure represents a complex, non-thermal oxidative challenge that enables investigation of cross-tolerance and adaptive responses in fungi exhibiting high resistance to conventional stressors.

Based on the information presented, the objectives of this study are: (i) characterize *Paecilomyces* species sampled from juice processing environments and spoiled products using multilocus phylogenetic analysis; (ii) assess strain-level variability in thermal tolerance among *Paecilomyces* isolates; (iii) investigate fungal responses to a non-thermal oxidative stressor using direct cold plasma exposure.

## Materials and methods

### Selection of *Paecilomyces* strains

*Paecilomyces* strains selected for this study were previously isolated by Margalho et al. (2025). These came from a non-carbonated orange juice processing line located in the “Citrus Belt,” in the Southern region of São Paulo State, Brazil. Twelve isolates were selected based on their sampling source and prior evaluations of sanitizer resistance and surface adhesion potential, as described by Margalho et al. (2025). Strains AC 66 and AC 67 were recovered from clean-in-place (CIP) water used after cleaning the juice storage tank. Strains AC 62, AC 63, AC 75, and AC 111 were isolated from the distribution nozzle of the feeder positioner. Strains AC 61 and AC 103 were obtained directly from pasteurized, spoiled juice prior to commercialization, while strains AC 72, AC 101, AC 102, and AC 110 were isolated from pasteurized, spoiled orange juice collected post-commercialization (Table 1).

**Table 1:** *Paecilomyces* strains used in this study and their origin within the orange juice processing line

Strains		Species	Origin		NCBI accession number			Reference
				β-tubulin			Calmodulin	
AC 61	*P. paravariotii*		Pasteurized juice*			PQ785546	PQ785562	Margalho et al. (2025).
AC 62	*P. paravariotii*		Filler nozzle			PQ785547	PQ785563	Margalho et al. (2025).
AC63	*P. paravariotii*		Filler nozzle			PQ785548	PQ785564	Margalho et al. (2025).
AC66	*P. paravariotii*		Clean in place water (CIP)			PQ785549	PQ785565	Margalho et al. (2025).
AC 67	*P. paravariotii*		Clean in place water (CIP)			PX213453	PX213454	This study
AC72	*P. paravariotii*		Pasteurized juice**			PQ785550	PQ785566	Margalho et al. (2025).
AC75	*P. paravariotii*		Filler nozzle			PQ785551	PQ785567	Margalho et al. (2025).
AC 101	*P. paravariotii*		Pasteurized juice**			PV388901	PV388905	This study
AC 102	*P. variotii*		Pasteurized juice**			PQ785554	PQ785570	Margalho et al. (2025).
AC103	*P. paravariotii*		Pasteurized juice*			PV388902	PV388906	This study
AC110	*P. lecythidis*		Pasteurized juice**			PV388903	PV388907	This study
AC 111	*P. dactylethromorphus*		Filler nozzle			PV388904	PV388908	This study

*Strains isolated from an orange juice sample collected after pasteurization, in the processing line

**Strains isolated from an orange juice sample collected from the market following a product recall due to fungal spoilage

### Characterization of *Paecilomyces* isolates

#### Identification of *Paecilomyces* species

The isolates were cultured on potato dextrose agar (PDA) for 5 days at 26 °C in a BOD incubator. Genomic DNA was then extracted using the Easy-DNA™ kit (Invitrogen, USA), following the manufacturer’s instructions.

For *BenA* locus, each reaction mixture contained 17.3 µL of ultrapure water, 2.5 µL of Buffer, 1 µL of MgCl₂ solution (25 mM), 2 µL of dNTP mix (10 mM), 0.5 µL of each forward and reverse primer (20 µM), 0.2 µL of GoTaq^®^ DNA Polymerase (5 U/µL, Promega), and 1 µL of genomic DNA (50–100 ng). For the *CaM* locus, the reaction mixture consisted of 13 µL of ultrapure water, 4 µL of Flexi Buffer, 3 µL of MgCl₂ solution (25 mM), 2 µL of dNTP mix (10 mM), 0.8 µL of each forward and reverse primer (20 µM), 0.3 µL of GoTaq^®^ DNA Polymerase (5 U/µL), and 1 µL of genomic DNA (50–100 ng). The primer pairs Bt2a/Bt2b and CMD5/CMD6 were used for amplification of the *BenA* and *CaM* loci, respectively (Glass and Donaldson 1995; Hong et al. 2006). Afterward, the following cycling conditions were applied: initial denaturation at 95 °C for 1 min; 35 cycles of denaturation at 94 °C for 1 min, annealing at 59 °C for *BenA* or 56 °C for *CaM* for 45 s, and extension at 72 °C for 1 min; followed by a final extension at 72 °C for 5 min. The reactions were carried out in a Veriti™ Thermal Cycler (Applied Biosystems, USA).

After PCR reactions, the fragments were purified using the ExoSAP-IT™ Express kit (Thermofisher, USA) and sent to the Central Laboratory for High-Performance Technologies in Life Sciences (LaCTAD) at the State University of Campinas (UNICAMP) for Sanger sequencing. The chromatograms were visualized in Geneious (Version 9). Multiple alignment was conducted using the ClustalW plug-in, and adjustments were made manually (Savi et al. 2018). The sequences of the strains were deposited in the NCBI database (Table 1).

Afterward, unweighted parsimony analysis was performed on the concatenated *BenA* and *CaM* dataset using paup v.4.0b10 (Swofford 2002), with a heuristic search option with 1000 random addition sequences and tree bisection reconnection branch swapping. Gaps were treated as missing data. The consistency index (CI) and the retention index (RI) were calculated to indicate the amount of homoplasy present. Clade stability was determined with maximum parsimony bootstrap proportions (MPBS) in paup v. 4.0b10, using 1000 heuristic search replications with random sequence addition (Tralamazza et al. 2021). The phylogenetic trees were visualized using FigTree v. 1.4 (Rambaut 2012). Furthermore, Bayesian analysis was performed using MrBayes (NGPhylogeny.com) to estimate Bayesian Posterior Probabilities (BPP) at consensus nodes. For this analysis, 1,000,000 trees were generated using 8 chains and 2 independent runs, applying the K2 + G + I (Kimura 2-Parameter + Gamma + Invariant Sites) model. The best-fitting substitution model was previously determined by Maximum Likelihood (ML) analysis in MEGA 12, based on the lowest Bayesian Information Criterion (BIC) value. Phylogenetic trees were visualized using FigTree v.1.4 (University of Edinburgh, Edinburgh, UK). *Monascus floridanus* was used as a suitable outgroup (Visagie et al. 2024). The dataset used for phylogenetic analysis was based on Visagie et al. (2024), with the sequences retrieved from the NCBI database.

#### Identification of MAT alleles in *Paecilomyces* strains

DNA extraction was conducted as previously described (item 2.2.1). PCR reactions for idiomorph identification were performed separately, following the protocol described by Houbraken et al. (2008). The primer sequences used were MAT1-1F1-VarSp (5’-TATGCCTCCTGGTGAGCTGG-3’) and MAT1-R2-VarMar (5’-ATCCCRGAYTTSGYCTTCTG-3’), which amplify a 334 bp fragment for mating type 1 (MAT1-1); and MAT2-F1Paec (5’-AYCAYCAYCCKATYGTCAAAGC-3’) and MAT2-R1Paec (5’-GYTTGCGYTTATCTSCTCYGC-3’), which amplify a 188 bp fragment for mating type 2 (MAT1-2) (Houbraken et al. 2008).

MAT alleles were identified using primers targeting MAT1-1 and MAT1-2 genes to assess whether there was a balanced proportion of idiomorphs within their ecological niche and, consequently, to evaluate the potential for sexual reproduction between strains of the same species; given that *P. variotii* and *P. paravariotii* are heterothallic. For *P. lecythidis* and *P. dactylethromor*p*hus*, the presence of these alleles was assessed solely to complement the current study, as available data for these species remain limited (Cairns et al. 2019; Seekles et al. 2023; van den Brule et al. 2020b).

### Growth conditions and conidial harvesting

For the following experiments the strains were grown in MEA (agar, 15 g; malt extract, 15 g; D-glucose, 15 g; peptone, 1 g; and distilled water, 1000mL) and incubated for 7 days at 26 °C. The tests that were performed using conidia suspensions were prepared as described by van den Brule et al. (2022). After growth, a solution of 20mL of PBS (Phosphate-Buffered Saline, pH 7) with 0.02% Tween-80 was added to the cultures (Seekles et al. 2023). With the aid of a disposable inoculation loop the culture was scraped and filtered through double layered gauze so that the hyphae would be retained. The suspensions were standardized using a Neubauer chamber, obtaining a final concentration of 2 × 10^7^ conidia/mL.

### Thermal resistance of *Paecilomyces* strains

Conidia of the 12 isolated strains of *Paecilomyces* spp. were evaluated for their thermal resistance to assess their potential survival throughout the orange juice processing line. The conidial suspension was prepared as described in Sect. 2.2.3.

To ensure uniform heat transfer throughout the spore suspension, 10 heparin-free borosilicate glass capillaries (internal diameter, 1 mm; external diameter, 1.5 mm; length, 75 mm; wall thickness, 0.1–0.2 mm) were used for each biological replicate and filled with the standardized spore suspension. The combined volume obtained from the 10 capillaries was sufficient for subsequent serial dilution, enabling the transfer of 100 µL aliquots into 900 µL of peptone water. After filling, the capillaries were flame-sealed and immersed in a circulating water bath previously equilibrated to the target temperature for 10 min to allow thermal stabilization (Alvarenga et al. 2021).

All tests were carried out in biological triplicates, and after heat treatment, the samples were serially diluted up to 10^− 3^ in peptone water (1%, v/v). Afterward, a 100 µL of inoculum was plated on MEA (Malt Extract Agar) in duplicate and incubated at 26 °C for up to seven days to determine the concentration of viable conidia; expressed as log _10_ of colony-forming units (CFU)/mL. The spore suspension without heat treatment was used as a positive control. For the negative control, only phosphate-buffered saline was used.

#### Screening for thermal resistance of *Paecilomyces* strains

The thermal resistance screening was carried out according to van den Brule et al. (2020a). Temperatures of 58 and 59 °C were selected for screening, as values within this range have previously been shown to discriminate differences in microbial survival (van den Brule et al. 2020a). Furthermore, temperature variations of only 1–2 °C can result in significant differences in logarithmic reduction, as demonstrated by Sant’Ana et al. (2009). The suspensions were added to the capillaries by pressure and then heat-treated at 58 and 59 °C for 10 min. Samples were instantly transferred to an ice bath to stop the process. After breaking the capillaries, decimal dilutions of all the samples up to 10^− 3^ were carried out, including the positive and negative controls. All plates were incubated and counted for between 3 and 5 days after heat treatment. The results were plotted as a heatmap with dendrograms obtained by hierarchical clustering based on Euclidean distance and Ward’s method, allowing the identification of patterns regarding the heat resistance of the studied strains(Corrêa et al. 2023; Murtagh and Legendre 2014). Additionally, Tukey’s multiple comparison test (*p* < 0.05) was applied to the data obtained at the highest temperature (59 °C) for a structured and reproducible selection of thermal resistance levels.

#### Thermal inactivation curves

After the screening for thermal resistance, six strains were selected to perform the inactivation curves and the subsequent experiments, two with low, intermediate, and high resistance to the thermal treatment applied. The thermal inactivation curves were based on Garcia et al. (2019); van den Brule et al. (2020a); Brule et al. (2020b). This temperature is widely used as a discriminatory condition in studies of conidial heat resistance, allowing robust quantitative comparisons among highly heat-resistant strains. It also lies close to the upper survival threshold reported for many eukaryotic microorganisms, although thermal tolerance may vary among genera, species, isolates, and substrates (Tuomela et al. 2000). The suspensions were heat-treated at 60 °C for 1, 3, 5, 7, 10, 15, 20, 30, 45, 60, 90, 120, 180, 200, 240 and 300 min. After each period, 10 capillaries (duplicates) were removed and instantly immersed in an ice bath to stop for thermal inactivation. After incubation for 3–5 days at 26 °C, cultures containing between 1 and 150 colonies were selected for counting (CFU/mL-1).

Similar to the approach described by Belloli et al. (2022) the 1 *D* value was derived from the *δ* parameter. The GInaFiT software (Geeraerd and Van Impe Inactivation Model Fitting Tool, version 1.7), which is compatible with Microsoft^®^ Excel, was used for model fitting (available at https://cit.kuleuven.be/biotec/software/GinaFit). The Weibull model was selected as the most appropriate for fitting the thermal inactivation data, as supported by previous studies (Bevilacqua et al. 2015; Zhang et al. 2018) This selection was based on the shape of the inactivation curves, which exhibited a characteristic “tailing” behavior. The model parameters, *δ* (scale parameter) and *p* (shape parameter), were obtained from the curve fitting. To enhance the stability of the *δ* estimates and improve the overall fit (correlation coefficient), the average *p*-value derived from all individual strain analyses was fixed for subsequent modeling, following the approach proposed by Mafart et al. (2002). The initial equation of the Weibull model is given by:

1 $$\log\;N=\log\;No-(\frac t\delta)p$$

Where: N is the microbial population at time *t*, *N₀* is the initial population, *t* is time (min), *δ* is the time required for the first decimal reduction, and *p* is the shape parameter. The parameter *δ* indicates the time interval necessary for microbial inactivation, while the shape parameter *p* characterizes the curvature of the survival curve over time. When *p* = 1, the microbial inactivation follows a log-linear behavior; when *p* < 1, the population becomes more resistant over time (concave curve); and when *p* > 1, the population becomes more sensitive (convex curve). Standard deviation and the coefficient of determination (R²) were calculated to evaluate the consistency and goodness-of-fit of the model (Souza et al. 2017).

The original Weibull model equation was rearranged to calculate the time required for a 1-log reduction in the microbial population (i.e., log *N* − log *N*₀ = −1):

2 $$1=-(t/\delta)p\rightarrow t_{5\delta}=5(\frac1p)\delta$$

It is important to note that this allows for the calculation of the time required to achieve any desired log reduction, accounting for non-linear inactivation kinetics (Santos et al.,^2018^).

### Stress responses of *Paecilomyces* strains to direct cold plasma exposure based on gliding arc plasma jet (GAPJ) system

**Fig. 1:**
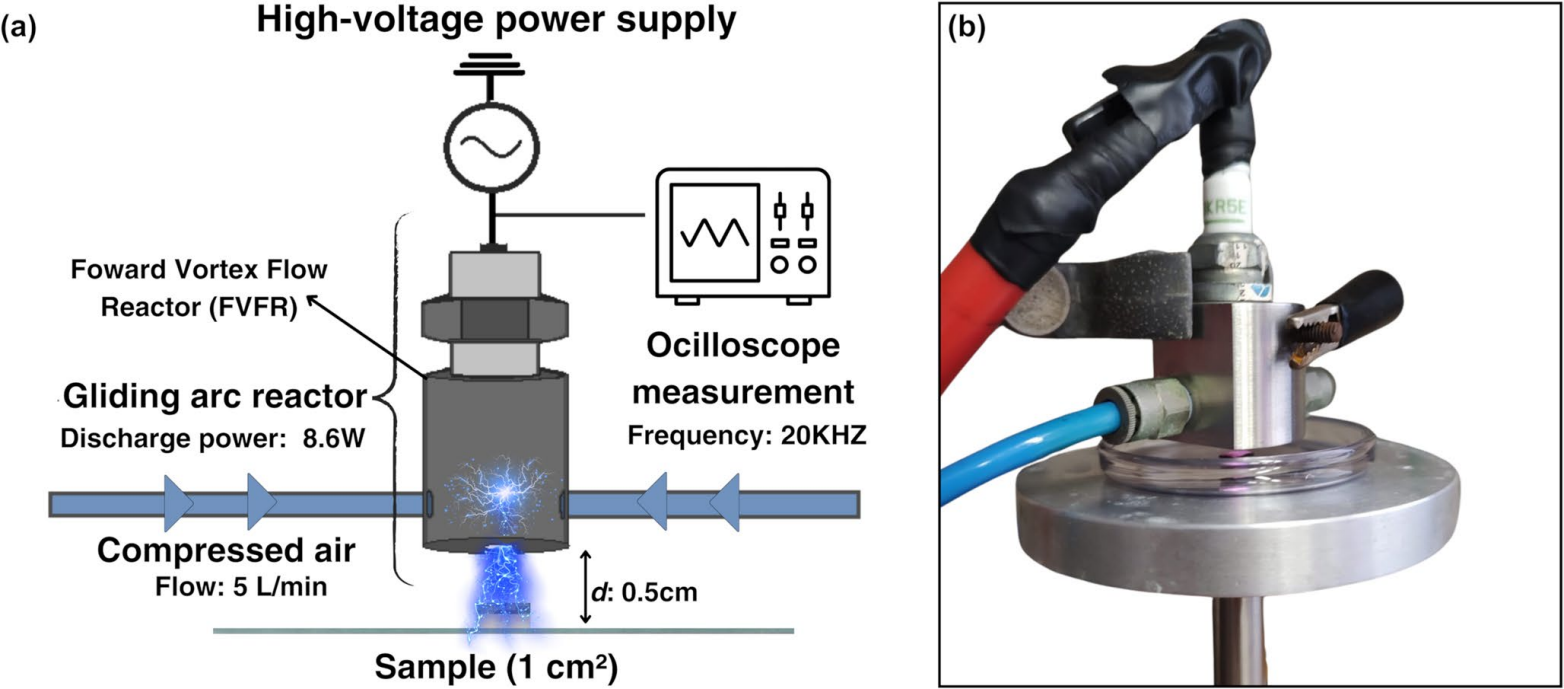
Experimental setup of the Gliding Arc Plasma Jet (GAPJ). (**a**) Schematic diagram showing the power supply, monitoring equipment, and reactor components. (**b**) Image of the reactor during cold plasma action

The GAPJ system was designed following the methodologies described by Chiappim et al. (2023) and Pereira et al. (2024). The setup is shown in Fig. 1, and all GAPJ treatments were conducted at the Plasma and Processes Laboratory (LPP) of the Technological Institute of Aeronautics (ITA). Cold plasma was generated in a reverse vortex flow reactor (FVFR) using atmospheric air at a constant flow rate of 5 L min⁻¹, monitored with a rotameter (Omega, 0–10 L min⁻¹). The discharge was powered by a high-voltage transformer (Linsa, São Paulo, Brazil) operating at 2.6 kV and 20 kHz, yielding a peak-to-peak voltage of 3.7 kV, a peak-to-peak current of 35 mA, and an average power of 8.6 W. A 1 kΩ high-voltage resistor was connected in series to protect the transformer from electrical arcs, and the output voltage of the plasma reactor was regulated using a Variac transformer (VARIAC, Cleveland, OH, USA).

The six strains selected in Sect. 2.3.1 were subjected to GAPJ treatment. Spore suspensions (2 × 10⁷ conidia mL⁻¹) were prepared according to Sect. 2.2.3, inoculated onto MEA and incubated for seven days at 26 °C. Agar plugs (0.7 cm²) were then excised and placed on glass slides for plasma exposure.

Preliminary experiments were conducted to optimize the cold plasma treatment conditions by evaluating the distance between the reactor outlet and the sample surface (2.5, 3, 5, and 10 mm) and the applied power (4, 8, and 10 W). Based on these assessments, a distance of 5 mm was selected, as it represented the shortest distance that did not result in a temperature > 45 °C. A power of 8.4 W was chosen to ensure antifungal activity and has previously been reported as suitable for food applications (Pereira et al. 2024). During plasma treatment, temperature was continuously monitored using a digital infrared thermometer and remained within the range of 40–45 °C (Fig. 1). Afterward, different exposure times were tested as follows: 2.5, 5, 10, 15, 20, 30, and 40 min.

For each strain treatment, and biological replicate, radial growth data were fitted by linear regression, from which slope and intercept values were obtained. The slope of the regression line was used to calculate the radial growth rate (mm/day), whereas the intercept was used as an indicator of the initial growth response, reflecting the delay in fungal adaptation after plasma exposure (lag phase) (Natarajan et al. 2022; Pereira et al. 2024). After exposure, the plugs were transferred to fresh MEA culture medium and incubated for 15 days at 26 °C. Untreated samples served as positive controls, while uninoculated medium was used as the negative control. All assays were performed in triplicate, and fungal growth was measured daily along the x and y axes with a digital caliper until maximum radial growth was achieved (90 mm diameter).

### Statistical analysis

Statistical analyses were performed using a combination of Microsoft^®^ Excel 365 (Microsoft, Redmond, WA, USA), R (version 4.4.1), and XLSTAT (Addinsoft^®^, Paris, France). Excel was used for data organization and preliminary visualization. R was employed to generate heatmaps, cluster strains based on thermal inactivation profiles, and perform statistical analyses including Tukey and Sidak multiple comparison tests (*p* < 0.05). Additional correlation analyses and Tukey HSD tests (*p* < 0.05) related to thermal inactivation and plasma-induced stress responses were conducted using XLSTAT.

## Results

### Molecular characterization of *Paecilomyces* strains

**Fig. 2:**
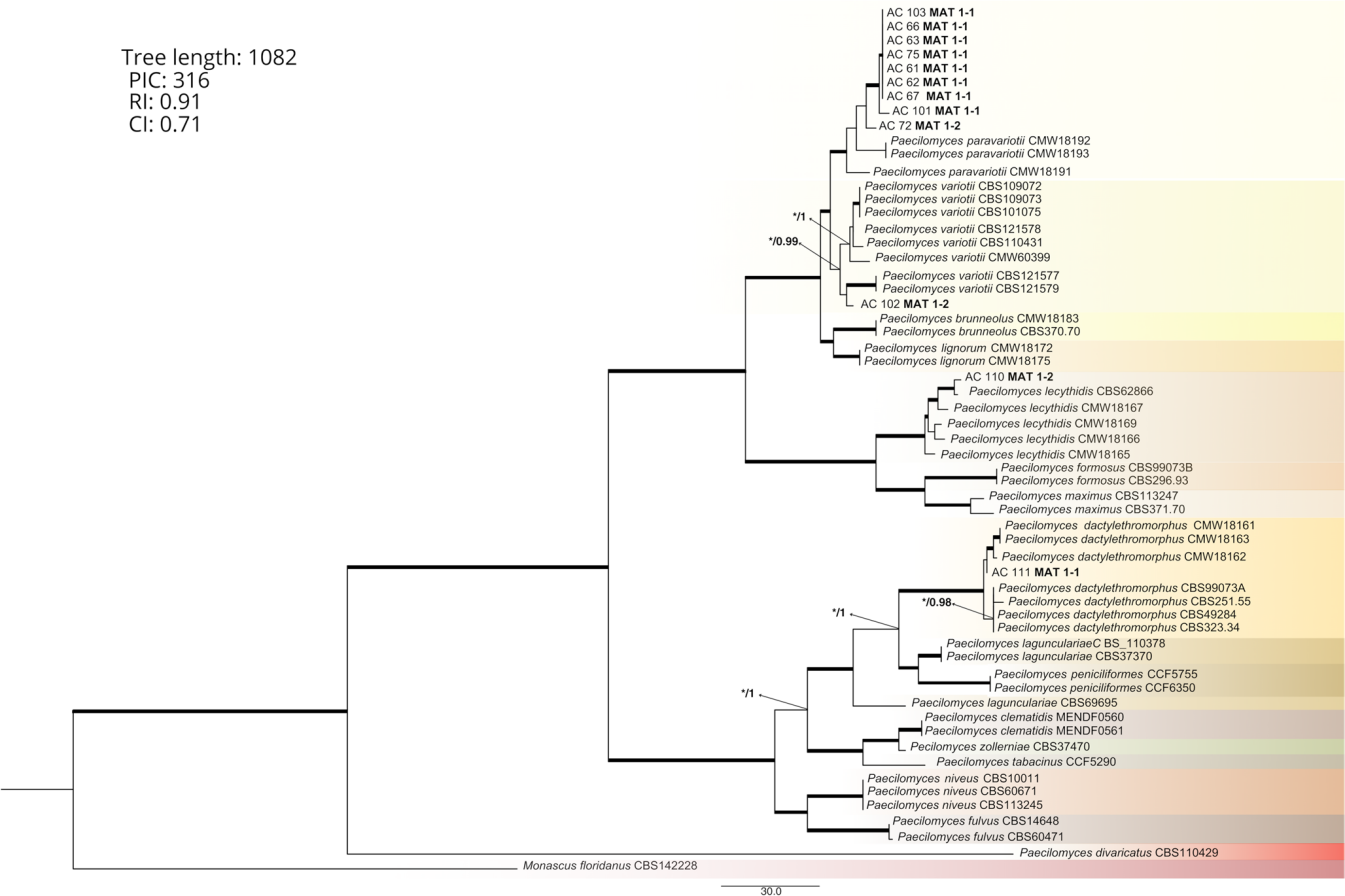
One of two most parsimonious trees inferred from beta-tubulin (*benA*) and calmodulin (*CaM*) combined loci, including one strain of *Paecilomyces variotti* and 9 of *P. paravariotii*. *Monascus floridanus* was used as outgroup. Branches designated in bold refer to Bootstrap intervals (BI) (1000 replications) greater than 70% and Bayesian posterior probabilities (BPP) greater than 0.95. Nodes where the BS value was less than 70% are represented by “*”, followed by the BPP value. PIC: parsimony-informative characters; CI: consistency index; RI: retention index

The concatenated *BenA* and *CaM* dataset consisted of 55 taxa, including the 12 isolates analyzed in this study and reference sequences retrieved from public databases, and comprised 1027 nucleotides with 316 parsimony-informative characters (PICs). The analysis resulted in two most parsimonious trees (CI = 0.71, RI = 0.91) (Fig. 2). Among the 12 isolates examined in the present study, nine were identified as *P. paravariotii*, a species recently described by Urquhart and Idnurm (2023), whereas one isolate was identified as *P. variotii* (AC 102), one as *P. dactylethromorphus* (AC 111), and one as *P. lecythidis* (AC 110).

Amplification of the mating-type genes in the selected strains yielded fragments of 334 bp for *MAT1-1* and 188 bp for *MAT1-2*, in accordance with the method described by (Houbraken et al. 2008). Among the *P. variotii* and *P. paravariotii* isolates, the distribution of idiomorphs was 80% *MAT1-1* and 20% *MAT1-2* (Fig. 2), indicating that sexual reproduction between these species under environmental conditions is unlikely. All reciprocal crosses between *P. paravariotii* strains (*MAT1-1* versus *MAT1-2*) were tested; however, no ascospore formation was observed (data not shown).

### Thermal resistance of *Paecilomyces* species

**Fig. 3:**
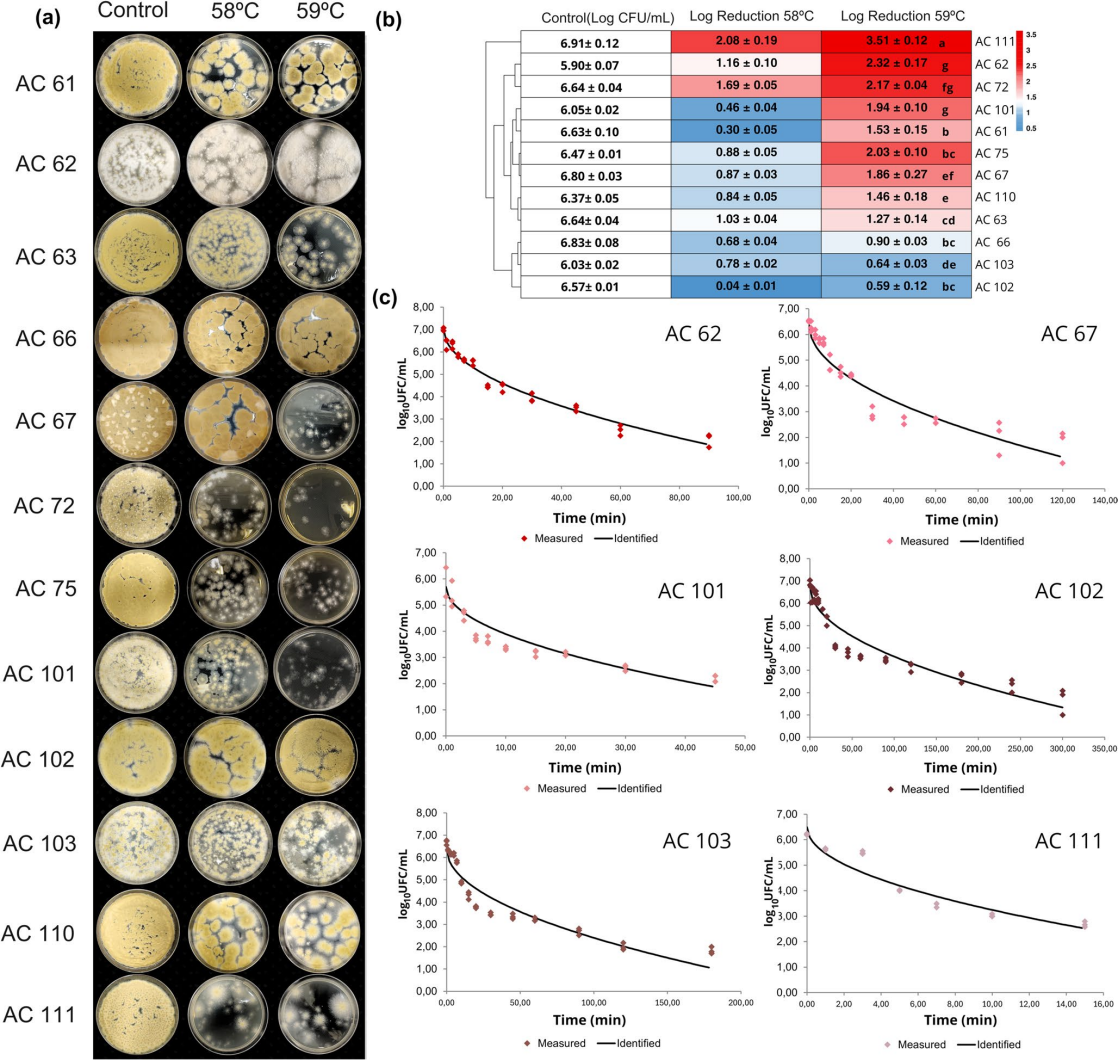
(**a**) Growth on MEA plates of the different strains (AC 62, AC 66, AC 67, AC 72, AC 75, AC 101, AC 102, AC 103, AC 110, and AC 111) after heat treatment at 58 °C and 59 °C, compared to the control (no treatment). The control was plated at dilution 10^− 4^, while the samples treated at 58 °C and 59 °C were plated at dilutions 10^− 3^ and 10^− 2^, respectively. (**b**) Heatmap with dendrograms obtained by hierarchical clustering analysis (Euclidean distance and Ward.D linkage method), illustrating the grouping of strains based on microbial reductions after heat treatment in relation to the untreated control (initial count). Mean values ± standard deviation are presented. On the color scale, red indicates greater logarithmic reduction compared to the control (lower heat resistance), while blue indicates lower logarithmic reduction (higher heat resistance). Different letters at 59 °C indicate statistically significant differences between strains according to Tukey’s test (*p* < 0.05). (**c**) Fitting of survival curves using the Weibull model (solid lines) to data from biological triplicates (points) using the average of experimental duplicates, demonstrating the variation in thermal inactivation parameters among the different strains studied. Counts were performed considering the detection limit of the method (1 log CFU/mL)

After treatment at 58 °C, reductions ranging from 0.04 log CFU/mL (strain AC 102) to 2.08 log CFU/mL (strain AC 111) were observed. At a temperature of 59 °C, reductions ranging from 0.59 log CFU/mL (strain AC 102) to 3.51 log CFU/mL (strain AC 111) were observed (Fig. 3a).

The heatmap revealed three distinct clusters based on thermal resistance profiles. Strains AC 66, AC 103, and AC 102 formed a group characterized by higher resistance to heat treatment, with reductions ranging from 0.59 to 1.27 log₁₀ CFU/mL. The second cluster included strains AC 61, AC 62, AC 63, AC 67, AC 72, AC 75, AC 101, and AC 110, which exhibited moderate resistance, with reductions between 1.46 and 2.32 log₁₀ CFU/mL. Strain AC 111 clustered separately due to its high sensitivity to heat, showing the greatest reduction in viability (3.51 log₁₀ CFU/mL) (Fig. 3b).

Based on these results, six strains were selected for thermal inactivation curve analysis at 60 °C. Strains AC 103 and AC 102 were chosen for their high thermal resistance, AC 67 and AC 101 for their intermediate resistance, and AC 62 and AC 111 for their greater sensitivity to thermal treatment (Fig. 3a and b).

The inactivation kinetics exhibited a nonlinear trend, with the Weibull model providing the best fit for subsequent statistical analyses. The average p-value was 0.52, indicating that the thermal inactivation curves followed a concave shape (*p* < 1). Most strains demonstrated a good model fit, with coefficients of determination (r²) exceeding 0.90. An exception was strain AC 62, which presented a lower r² value of 0.851. Biological variability among replicates was quantitatively expressed by the standard deviation of the δ parameter (Fig. 3c).

The thermal resistance profiles of the strains at 60 °C were consistent with those previously observed at 58 and 59 °C. As shown in Table 2, P. *variotii* AC 102 exhibited the highest thermal resistance, with a D₆₀ value exceeding 45 min as calculated according to the method described by (Punt et al. 2020). The *P. paravariotii* strains analyzed in this study showed intermediate resistance, with D₆₀ values ranging from 27.7 to 13.2 min. In contrast, strain AC 111, identified as *P. dactylethromorphus*, demonstrated the lowest thermal resistance, with a D₆₀ value of 4.20 min.

**Table 2:** Values ​​of *δ*_*60*_ (decimal reduction time in minutes at 60 °C), coefficient of determination (*R²*) and *D*_*60*_ (time required to reduce 90% of the population) for the different strains of *Paecilomyces* spp. after heat treatment

Strain	δ_60_^*^	*R* ^2^	D_60_^*^
AC 62	3.07 ± 0.18^c^	0.851	13.7
AC 67	4.50 ± 0.23^c^	0.910	19.88
AC 102	10.30 ± 0.23^a^	0.914	45.50
AC 101	3.37 ± 0.17^c^	0.917	14.89
AC103	6.28 ± 0.54^b^	0.903	27.74
AC 111	0.95 ± 0.05^d^	0.920	4.20

^*^The *δ*_*60*_ and *D*_*60*_ values ​​are expressed in minutes. The *δ*_*60*_ values ​​are presented as mean ± standard error (*n* = 3). Different letters indicate statistically significant differences between strains (*p* < 0.05) by Tukey’s test

### Stress responses of *Paecilomyces* strains to GAPJ

**Fig. 4:**
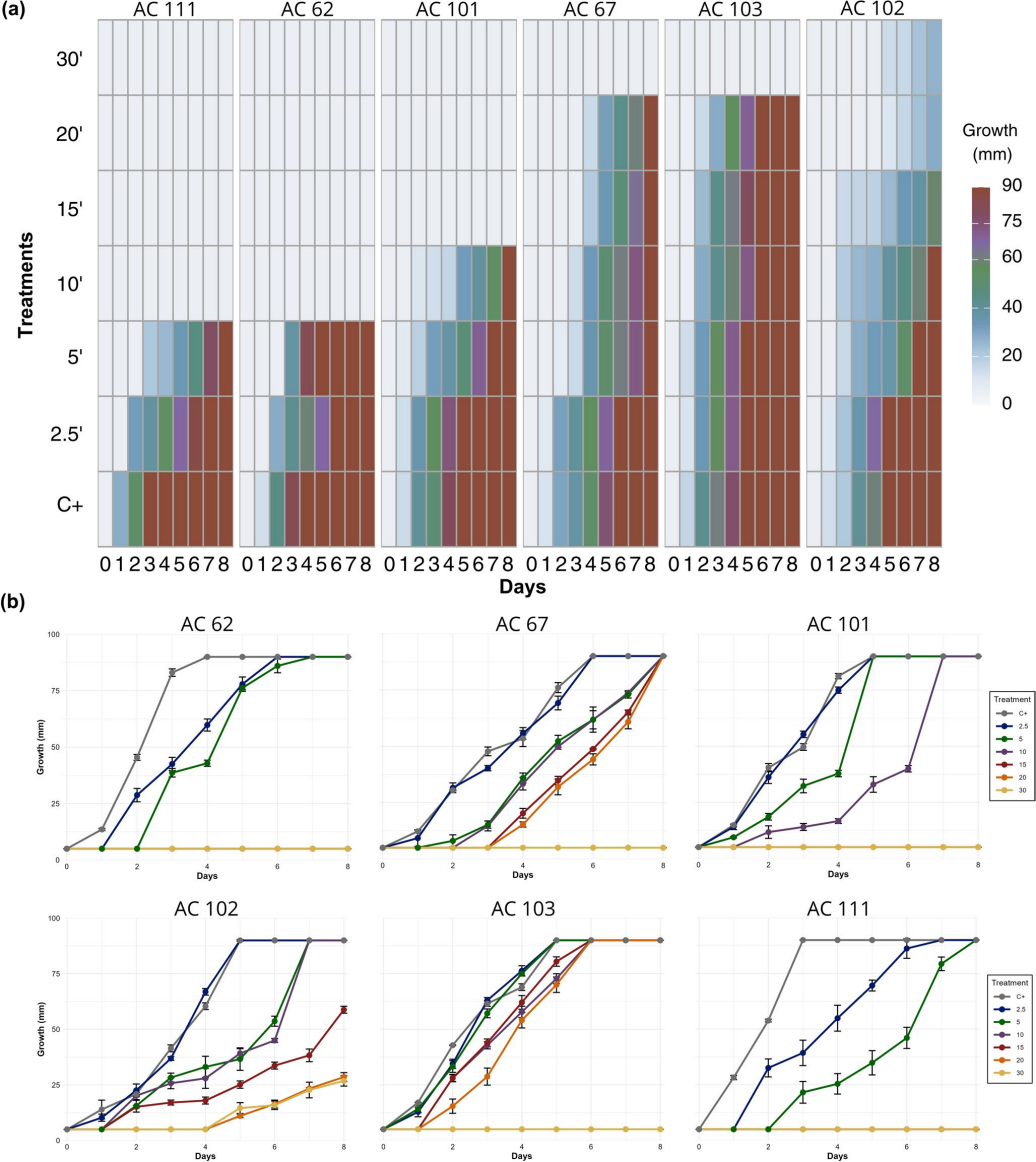
Radial growth of Paecilomyces strains after exposure to the gliding arc plasma jet (GAPJ). **(a)** Heatmap showing colony expansion over 8 days for strains AC 111, AC 62, AC 101, AC 67, AC 103, and AC 102 under different treatments: C+ (untreated control), 2.5, 5, 10, 15, 20, and 30 min. Columns correspond to observation days and rows to exposure times, with darker shades indicating greater growth. **(b)** Growth curves (mean ± standard error, *n* = 3) representing radial growth (mm) over 8 days for the same strains. The same color scheme was applied in both panels to denote GAPJ exposure times, ensuring visual correspondence between the heatmap and the curves

Based on radial growth measurements, *Paecilomyces* strains demonstrated comparable growth dynamics and resistance levels across the different GAPJ treatments. The strains exhibited similar resistance profiles when comparing heat treatment and cold plasma application. As shown in the heatmap (Fig. 4a), strain AC 102 was the most resistant, followed by strains AC 103 and AC 67. Strains AC 101, AC 62, and AC 111 showed progressively lower resistance levels.

Fungal growth rates were calculated by applying linear regressions to each replicate and treatment, with growth monitored during 8 days of incubation, as illustrated in Fig. 4b.

To understand the effect of GAPJ treatments (2.5, 5, 10, 15, 20, and 30 min), ANOVA followed by Sidak’s multiple comparison test (*p* < 0.05) was performed using the slope and intercept values calculated from the complete 0–8 day growth period for each strain. Analysis of slope values (Supplementary Figure S1a) indicated that AC 62 and AC 103 had higher overall growth slopes than the control, although both strains showed greater growth delay with increasing GAPJ exposure time. The calculations also demonstrated that the intercept value decreased as the treatment intensity increased, indicating that the more intense the plasma treatment, the slower the initial adaptation of the fungus, with AC 102 being the only one that resisted the 30-minutes treatment, with a delay of 4 days (Supplementary Figure S1b).

Most other strains showed a linear reduction in radial growth rate with increasing treatment intensity. For strain AC 102, no significant difference in growth rate was observed between the 20- and 30-minute treatments following the initial adaptation phase. Notably, the 2.5-minute treatment only resulted in a statistically significant delay in growth for the most sensitive strains (AC 111 and AC 62), both of which experienced a two-day delay in adaptation. The 5-minute treatment further reduced their growth rates, and complete growth inhibition was observed under the 10-minute treatment.

## Discussion

The current study provided new insights into the stress tolerance of *Paecilomyces* strains recovered from juice processing environments and spoiled products, revealing strain-level variability that is conserved across both thermal and non-thermal stressors. Multilocus phylogenetic analysis demonstrated the predominance of the recently described cryptic species *P. paravariotii*, alongside *P. variotii*,* P. lecythidis*, and *P. dactylethromorphus*, highlighting the diversity of food-associated *Paecilomyces* populations. Isolates exhibited markedly different responses to thermal stress, with *P. variotii* displaying higher heat tolerance, *P. paravariotii* showing intermediate resistance, and *P. dactylethromorphus* being more sensitive; in addition, a similar response was observed under non-thermal plasma-induced stress across the tested strains. Overall, this indicates that environmental selection pressures in juice processing environments may have induced *Paecilomyces* strains to resist both thermal and non-thermal stresses.

*Paecilomyces paravariotii* was first reported in association with orange juice by Margalho et al. (2025). As this species is closely related to *P. variotii*, reliance on limited genetic markers or morphological criteria may lead to misidentification in food systems. Accurate discrimination between these taxa requires multilocus analysis, and insufficient resolution may contribute to the underreporting of *P. paravariotii* in food products (Margalho et al. 2025; Urquhart and Idnurm 2023). In this context, the phylogenetic analysis based on *BenA* and *CaM* loci revealed that most of the strains clustered within the *P. paravariotii* clade, whereas AC 102, AC 110 and AC 111 were identified as *P. variotii*, *P. lecythidis* and *P. dactylethromor*p*hus*, respectively (Urquhart and Idnurm 2023; Visagie et al. 2024).

A few *Paecilomyces* species have been described in Brazilian studies; Among these, *P. variotii* is the most frequently reported, occurring in soil, food products, and clinical settings, with particular attention to its documented resistance to heat treatments and sanitizers (Ferreira et al. 2021; Garcia et al. 2019; Herrera Bravo de Laguna et al., 2015; Stefanello et al. 2020; Steiner et al. 2013). Other species and associated genera are primarily found in environmental and biotechnological contexts, including *P. formosus*, *P. parvisporus* and *Purpureocillium lilacinum*, observed in biodiversity surveys and agro-industrial residues (Andrade et al. 2023; Ferreira et al. 2016; Lopes et al. 2016).

Thermal and other stress-related resistance in fungi are often associated with ascospore formation; while some *Paecilomyces* species, such as *P. niveus*, are homothallic and more frequently encountered in their sexual stage, both *P. variotii* and *P. paravariotii* are heterothallic. In this study, the observed mating-type ratio was approximately 4:1 (MAT1-1: MAT1-2), suggesting a low probability of successful mating and ascospore formation in the processing environment. Furthermore, previous studies have reported the inability of *P. paravariotii* to produce ascospores in vitro (Urquhart and Idnurm 2023), which is consistent with the lack of observed sexual crossing in our *P. paravariotii* strains (data not shown). Considering this, the stress-related experiments were solely conducted on conidia obtained from our isolates; nevertheless, this is particular important, especially due to the well-known resistance of *P. variotii* conidia (van den Brule et al. 2020a).

Thermal resistance experiments identified strain AC 102, *P. variotii*, as the most resistant among those tested. Its thermal inactivation curve generated a high average value of *D*_60_ = 45.5 min (van den Brule et al. 2020a; Brule et al. 2020b, 2022). All *P. paravariotii* strains exhibited greater sensitivity to heat treatment; however, their D₆₀ values still fell within the range of 3.5 to 27.6 min, as previously reported for *P. variotii* by van den Brule et al. (2020b). This suggests that the thermal resistance profiles of these species are relatively similar. AC 110, identified as *P. lecythidis*, also displayed certain thermal resistance (van den Brule et al. 2022).

*P. variotii* warrants particular attention, as it is known to produce conidia with exceptionally thermal resistance and to undergo sexual reproduction in a heterothallic manner (Houbraken et al. 2008). Its ascospores can withstand a temperature of 85 °C for 45–75 min, depending on the food matrix (Ulusoy et al. 2022; van den Brule et al. 2022). Another important observation is that AC 103, the most resistant *P. paravariotii* strain, was recovered from orange juice collected immediately after pasteurization on the processing line (Margalho et al. 2025). This suggests that this strain, as well as other ones used in this study, may have been subjected to environmental selection pressure (Xiao et al. 2022), particularly due to high temperature exposure, which could have possibly contributed to the resistance to stress-related treatments, such as higher temperatures and cold plasma.

Finally, AC 111 (*P. dactylethromorphus*) was considered the most sensitive, with an average value of D_60_ = 4.2 min. This species is known to produce patulin (Moreira et al. 2018; Visagie et al. 2024); however, data on its thermal resistance remain scarce. Further studies are therefore appropriate, particularly given the food safety implications associated with patulin production.

Comparing the results of the thermal treatment with the cold plasma exposure, strain AC 102 (*P. variotii*) has also shown the highest resistance, maintaining growth even after a 30-minute exposure. Strain AC 103 (*P. paravariotii*) survived up to 20 min of treatment. In contrast, strain AC 111 (*P. dactylethromorphus*) exhibited high sensitivity to both thermal treatment and plasma-induced stress. This strain was isolated from the filler nozzle, suggesting that it may have originated from environmental contamination rather than from within the processing line itself. Consequently, it is likely that this isolate was not subjected to sustained selection pressure imposed by thermal processing, which may explain its increased sensitivity relative to strains recovered from post-processing environments.

*P. paravariotii* AC 62 exhibited reduced resistance to both heat and GAPJ treatments. Interestingly, this strain displayed distinct pigmentation, with a whitish or creamy appearance, which may reflect physiological differences. Indeed, melanin-deficient conidia have been shown to be more susceptible to UV-C (Seekles et al. 2021);in addition, pigment production by microorganisms, such as carotene and melanin are associated with cell protection against physical and chemical stressors; including reactive species and UV radiation generated during plasma treatments (Koch et al. 2023; Tong et al. 2023).

It is important to note that our GAPJ system operated at 40–45 °C, far below the thermal inactivation threshold of *Paecilomyces*, confirming that plasma-induced reactive oxygen and nitrogen species (RONS) not heat caused the effects. Its previously characterized emission spectrum includes intense N₂, OH and O bands (Chiappim et al. 2023; Pereira et al. 2024), supporting the high production of reactive species.

Cold plasma has emerged as an alternative non-thermal technology for controlling microbial contamination and promoting mycotoxin degradation in food systems, largely due to its reduced impact on nutritional and sensory attributes compared with thermal treatments (Laroque et al. 2022). However, limited information is available on microbial stress tolerance and on the potential toxicity of plasma-derived mycotoxin compounds, particularly when food matrices are considered as complex and distinct ecosystems, highlighting the need for further research in this area.

Stress adaptation in fungi is complex and associated with multiple mechanisms that include the recognition of environmental signals and regulation of cellular processes that mediate the response to different stresses (Xiao et al. 2022). For instance, the expression of chaperones, synthesis of antioxidants, accumulation of compatible solutes such as polyols and disaccharides are involved in these adaptative mechanisms (Punt et al. 2020; Seekles et al. 2023; van den Brule et al. 2022). Differences in conidial size have also been identified as a factor that may influence thermal resistance (van den Brule et al. 2020a). Moreover, repeated exposure to elevated temperatures or other stressors may induce these stress-response pathways, arising from both transcriptional changes and, over time, genetic variation, leading to adaptation to certain conditions and growth in environments that were previously unsuitable for the microorganism (Brown et al. 2017; Vogt, 2022).

## Conclusion

This study demonstrates that *Paecilomyces* strains associated with juice processing environments exhibited variability in stress tolerance that is largely similar across both thermal and non-thermal applications. The predominance of the *P. paravariotii*, that demonstrated different resistant profiles, together with the high resistance observed in *P. variotii*, highlights the importance of accurate species identification and strain-level assessment when evaluating fungal persistence in food-related environments.

The consistency between thermal resistance profiles and responses to plasma-induced oxidative stress suggests that food processing environments impose selection pressures that promote the adaptation of *Paecilomyces* strains to stressful conditions. Overall, these findings emphasize the role of environmental selection in shaping stress-adapted *Paecilomyces* populations and highlight the need to consider fungal ecology and adaptation when designing and evaluating food processing and preservation strategies.

## Supplementary Information

Below is the link to the electronic supplementary material.

Supplementary File 1 (PNG 912 KB)

Supplementary File 2 (DOCX 20.2 KB)

## Data Availability

The authors declare that the data supporting the findings of this study are available within the paper and its Supplementary Information files. Should any raw data files be needed in another format they are available from the corresponding author upon reasonable request.

## References

[CR1] Alvarenga VO, Gonzales-Barron U, do Prado Silva L, Cadavez V, Sant’Ana AS (2021) Using extended Bigelow meta-regressions for modelling the effects of temperature, pH, °Brix on the inactivation of heat-resistant moulds. Int J Food Microbiol. 10.1016/j.ijfoodmicro.2020.108985 10.1016/j.ijfoodmicro.2020.108985 33334619

[CR2] Andrade MC, Alves GSC, Fontes PR, Miller RNG, Filho EXF (2023) Hydrothermal treatment of Coffee residues for the production of pectinases by *Paecilomyces formosus*. Waste Biomass Valoriz. 10.1007/s12649-022-01981-w

[CR3] Belloli M, Cigarini M, Milesi G, Mutti P, Berni E (2022) Effectiveness of two UV-C light-emitting diodes (LED) systems in inactivating fungal conidia on polyethylene terephthalate. Innov Food Sci Emerg Technol 79:103050. 10.1016/J.IFSET.2022.103050

[CR4] Bernardi AO, Garcia MV, Copetti MV (2019) Food industry spoilage fungi control through facility sanitization. Curr Opin Food Sci. 10.1016/j.cofs.2019.07.006

[CR5] Bevilacqua A, Speranza B, Sinigaglia M, Corbo MR (2015) A focus on the death kinetics in predictive microbiology: benefits and limits of the most important models and some tools dealing with their application in foods. Foods 4(4):565–580. 10.3390/FOODS4040565 28231222 10.3390/foods4040565 PMC5224560

[CR6] Brown AJP, Cowen LE, di Pietro A, Quinn J (2017) Stress adaptation. Microbiol Spectr. 10.1128/MICROBIOLSPEC.FUNK-0048-2016 28721857 10.1128/microbiolspec.funk-0048-2016 PMC5701650

[CR7] Cairns TC et al (2019) A quantitative image analysis pipeline for the characterization of filamentous fungal morphologies as a tool to uncover targets for morphology engineering: a case study using aplD in *Aspergillus niger*. Biotechnol Biofuels. 10.1186/s13068-019-1473-0 31223339 10.1186/s13068-019-1473-0 PMC6570962

[CR8] Carvalho TB et al (2024) Preventing fungal spoilage from raw materials to final product: innovative preservation techniques for fruit fillings. Foods 13(17):2669. 10.3390/foods13172669 39272437 10.3390/foods13172669 PMC11394069

[CR9] Cherif MM, Assadi I, Khezami L, Ben Hamadi N, Assadi AA, Elfalleh W (2023) Review on recent applications of cold plasma for safe and sustainable food production: principles, implementation, and application limits. Appl Sci. 10.3390/app13042381

[CR10] Chiappim W, de Paula Bernars V, Almeida NA, Pereira VL, Bragotto APA, Cerqueira MBR, Furlong EB, Pessoa R, Rocha LO (2023) Effect of Gliding Arc Plasma Jet on the mycobiota and deoxynivalenol levels in naturally contaminated barley grains. Int J Environ Res Public Health. 10.3390/ijerph20065072 36981981 10.3390/ijerph20065072 PMC10049212

[CR11] Corrêa JMM, de Oliveira MLG, de Souza PG, Filho PMS, de Macedo AN, Faria AF (2023) Optimization of the first extraction protocol for metabolomic studies of *Brucella abortus*. Braz J Microbiol 54:2383–2392. 10.1007/S42770-023-01001-6 37209273 10.1007/s42770-023-01001-6 PMC10484873

[CR12] Dabija A, Afloarei C, Ștefania, Dabija D, Chetrariu A (2025) Conventional and Innovative Methods for Reducing the Incidence of Listeria monocytogenes in Milk and Dairy Products. Applied Sciences 2025, Vol. 15, Page 6580 15, 6580. 10.3390/APP15126580

[CR13] Davies CR, Wohlgemuth F, Young T, Violet J, Dickinson M, Sanders JW, Vallieres C, Avery SV (2021) Evolving challenges and strategies for fungal control in the food supply chain. Fungal Biol Rev 36:15–26. 10.1016/J.FBR.2021.01.003 34084209 10.1016/j.fbr.2021.01.003 PMC8127832

[CR14] Desai M, Chandel A, Chauhan OP, Semwal AD (2024) Uses and future prospects of cold plasma in agriculture. Food and Humanity. 10.1016/j.foohum.2024.100262

[CR15] Dijksterhuis J, Meijer M, van Doorn T, Samson R, Rico-Munoz E (2018) Inactivation of stress-resistant ascospores of Eurotiales by industrial sanitizers. Int J Food Microbiol 285:27–33. 10.1016/J.IJFOODMICRO.2018.06.018 30015260 10.1016/j.ijfoodmicro.2018.06.018

[CR16] Ferreira GF, Freitas TM, Gonçalves CL, Mendes JF, Vieira JN, Villareal JP, Nascente PS (2016) Antiparasitários: Teste in vitro contra fungos nematófago. Brazilian J Biology 76. 10.1590/1519-6984.05615 10.1590/1519-6984.05615 27224732

[CR17] Ferreira MA, Costa RAF, Bispo A, Choupina AB, Evangelista-Barreto NS, Carvalho C, Estevinho MLMF, Sodré G (2021) Diversidade da microbiota de fungos da própolis in natura / Diversity of in natura propolis fungal microbiota. Brazilian Journal of Animal and Environmental Research. 10.34188/bjaerv4n4-122

[CR18] Frisvad JC (2018) A critical review of producers of small lactone mycotoxins: patulin, penicillic acid and moniliformin. World Mycotoxin J 11:73–100. 10.3920/WMJ2017.2294

[CR19] Garcia MV, Bernardi AO, Parussolo G, Stefanello A, Lemos JG, Copetti MV (2019) Spoilage fungi in a bread factory in Brazil: diversity and incidence through the bread-making process. Food Res Int 126:108593. 10.1016/J.FOODRES.2019.108593 31732034 10.1016/j.foodres.2019.108593

[CR20] Glass NL, Donaldson GC (1995) Development of primer sets designed for use with the PCR to amplify conserved genes from filamentous ascomycetes. Appl Environ Microbiol 61(4):1323–1330. 10.1128/AEM.61.4.1323-1330.1995 7747954 10.1128/aem.61.4.1323-1330.1995 PMC167388

[CR21] Herrera Bravo de Laguna I, Toledo Marante FJ, Mioso R (2015) Enzymes and bioproducts produced by the ascomycete fungus *Paecilomyces variotii*. J Appl Microbiol. 10.1111/jam.12934 26274842 10.1111/jam.12934

[CR22] Hong SB, Cho HS, Shin HD, Frisvad JC, Samson RA (2006) Novel *Neosartorya* species isolated from soil in Korea. Int J Syst Evol Microbiol 56(2):477–486. 10.1099/IJS.0.63980-0 16449461 10.1099/ijs.0.63980-0

[CR23] Hosoya K, Nakayama M, Tomiyama D, Matsuzawa T, Imanishi Y, Ueda S, Yaguchi T (2014) Risk analysis and rapid detection of the genus *Thermoascus*, food spoilage fungi. Food Control. 10.1016/j.foodcont.2013.12.021

[CR24] Houbraken J, Samson RA, Frisvad JC (2005) Byssochlamys: Significance of heat resistance and mycotoxin production, in: Advances in Experimental Medicine and Biology. 10.1007/0-387-28391-9_14 10.1007/0-387-28391-9_14 16408604

[CR25] Houbraken J, Varga J, Rico-Munoz E, Johnson S, Samson RA (2008) Sexual reproduction as the cause of heat resistance in the food spoilage fungus *Byssochlamys spectabilis* (anamorph *Paecilomyces variotii*). Appl Environ Microbiol. 10.1128/AEM.01761-07 18192427 10.1128/AEM.01761-07 PMC2258620

[CR26] Jafarzadeh S, Hadidi M, Forough M, Nafchi AM, Mousavi Khaneghah A (2023) The control of fungi and mycotoxins by food active packaging: a review. Crit Rev Food Sci Nutr. 10.1080/10408398.2022.2031099 35089844 10.1080/10408398.2022.2031099

[CR27] Kaur S, Kumar Y, Singh V, Kaur J, Panesar PS (2024) Cold plasma technology: reshaping food preservation and safety. Food Control. 10.1016/j.foodcont.2024.110537

[CR28] Koch SM, Freidank-Pohl C, Siontas O, Cortesao M, Mota A, Runzheimer K, Jung S, Rebrosova K, Siler M, Moeller R, Meyer V (2023) *Aspergillus niger* as a cell factory for the production of pyomelanin, a molecule with UV-C radiation shielding activity. Front Microbiol. 10.3389/fmicb.2023.1233740 37547691 10.3389/fmicb.2023.1233740 PMC10399693

[CR29] Laroque DA, Seó ST, Valencia GA, Laurindo JB, Carciofi BAM (2022) Cold plasma in food processing: design, mechanisms, and application. J Food Eng. 10.1016/j.jfoodeng.2021.110748

[CR30] Lemos JG, Silva LP, Mahfouz MAAR, Cazzuni LAF, Rocha LO, Steel CJ (2025) Use of dielectric-barrier discharge (DBD) cold plasma for control of bread spoilage fungi. Int J Food Microbiol. 10.1016/j.ijfoodmicro.2024.111034 39731988 10.1016/j.ijfoodmicro.2024.111034

[CR31] Lim S, Bijlani S, Blachowicz A, Chiang YM, Lee MS, Torok T, Venkateswaran K, Wang CCC (2021) Identification of the pigment and its role in UV resistance in *Paecilomyces variotii*, a Chernobyl isolate, using genetic manipulation strategies. Fungal Genet Biol. 10.1016/j.fgb.2021.103567 33989788 10.1016/j.fgb.2021.103567

[CR32] Lopes A, Vitorino L, Castro C, Nascimento J, Souchie E (2016) Primeiro relato da ocorrência de *Paecilomyces formosus* e *Paecilomyces parvisporus* no Brasil. Revista Brasileira de Biociências, p 14

[CR34] Luangsa-Ard J, Houbraken J, van Doorn T, Hong SB, Borman AM, Hywel-Jones NL, Samson RA (2011) *Purpureocillium*, a new genus for the medically important *Paecilomyces lilacinus*. FEMS Microbiol Lett 321:141–149. 10.1111/j.1574-6968.2011.02322.x 21631575 10.1111/j.1574-6968.2011.02322.x

[CR33] Luangsa-ard JJ, Hywel-Jones NL, Samson RA (2004) The polyphyletic nature of *Paecilomyces* sensu lato based on 18S-generated rDNA phylogeny. Mycologia 96:773–780. 10.1080/15572536.2005.11832925 21148898 10.1080/15572536.2005.11832925

[CR35] Mafart P, Couvert O, Gaillard S, Leguerinel I (2002) On calculating sterility in thermal preservation methods: application of the Weibull frequency distribution model. Int J Food Microbiol 72:107–113. 10.1016/S0168-1605(01)00624-9 11843401 10.1016/s0168-1605(01)00624-9

[CR36] Margalho LP, Martins CS, Almeida NA, Carusi J, Mahfouz MAAR, Sant’Ana AS, Nascimento MS, de Oliveira Rocha L (2025) Fungi associated with orange juice production and assessment of adhesion ability and resistance to sanitizers. Int J Food Microbiol. 10.1016/j.ijfoodmicro.2024.111035 39731990 10.1016/j.ijfoodmicro.2024.111035

[CR37] Meneses-Espinosa E, Gálvez-López D, Rosas-Quijano R, Adriano-Anaya L, Vázquez-Ovando A (2024) Advantages and disadvantages of using emerging technologies to increase postharvest life of fruits and vegetables. Food Rev Int. 10.1080/87559129.2023.2212061

[CR38] Mirza Alizadeh A, Hashempour-Baltork F, Mousavi Khaneghah A, Hosseini H (2021) New perspective approaches in controlling fungi and mycotoxins in food using emerging and green technologies. Curr Opin Food Sci. 10.1016/j.cofs.2020.12.006

[CR39] Monpierre L, Aït-Ammar N, Valsecchi I, Normand AC, Guitard J, Riat A, Huguenin A, Bonnal C, Sendid B, Hasseine L, Raberin H, Dehais M, Ranque S, Hennequin C, Piarroux R, Dannaoui E, Botterel F (2022) Species identification and in vitro antifungal susceptibility of Paecilomyces/Purpureocillium species isolated from clinical respiratory samples: a multicenter study. J Fungi. 10.3390/jof8070684 10.3390/jof8070684 PMC9321559 35887446

[CR40] Moreira DC, Oliveira MME, Borba CM (2018) Human pathogenic *Paecilomyces* from food. Microorganisms. 10.3390/microorganisms6030064 29976858 10.3390/microorganisms6030064 PMC6164242

[CR41] Moreno-Gavíra A, Huertas V, Diánez F, Santos M, Sánchez-Montesinos B (2020) *Paecilomyces* and its importance in the biological control of agricultural pests and diseases. Plants. 10.3390/plants9121746 33321854 10.3390/plants9121746 PMC7763231

[CR42] Murtagh F, Legendre P (2014) Ward’s hierarchical agglomerative clustering method: which algorithms implement ward’s criterion? J Classif 31:274–295. 10.1007/s00357-014-9161-z

[CR44] Natarajan S, Balachandar D, Senthil N, Paranidharan V (2022) Interaction of water activity and temperature on growth, gene expression, and aflatoxin B1 production in *Aspergillus flavus* on Indian senna (*Cassia angustifolia* Vahl.). Int J Food Microbiol 361(6):109457. 10.1016/j.ijfoodmicro.2021.109457 34742145 10.1016/j.ijfoodmicro.2021.109457

[CR45] Neuenfeldt NH, Silva LP, Pessoa RS, O Rocha L (2023) Cold plasma technology for controlling toxigenic fungi and mycotoxins in food. Curr Opin Food Sci. 10.1016/j.cofs.2023.101045

[CR46] Patil V, Shams R, Dash KK (2024) Cold plasma pretreatment for transforming fruit and vegetable waste: a comprehensive review. Future Foods. 10.1016/j.fufo.2024.100400

[CR47] Peloso M, Minkoumba Sonfack G, Prizio I, Baraldini Molgora E, Pedretti G, Fedrizzi G, Caprai E (2024) Climate effects on ergot and ergot alkaloids occurrence in Italian wheat. Foods. 10.3390/foods13121907 38928849 10.3390/foods13121907 PMC11202928

[CR48] Perdomo H, García D, Gené J, Cano J, Sutton DD, Summerbell R, Guarro J (2013) *Phialemoniopsis*, a new genus of Sordariomycetes, and new species of *Phialemonium* and *Lecythophora*. Mycologia 105:398–421. 10.3852/12-137 23099515 10.3852/12-137

[CR49] Pereira VL, Caramês E, Almeida NA, Chiappim W, Pessoa RS, Petraconi Filho G, Rocha L (2024) Gliding arc plasma jet for inhibiting mycotoxin production and apple brown rot by *Alternaria alternata*. Food Control 155:110108. 10.1016/J.FOODCONT.2023.110108

[CR50] Pitt J.I., Hocking A.D. (2022) Fungi and Food Spoilage: Fourth Edition. Fungi Food Spoilage: Fourth Ed 1–645. 10.1007/978-3-030-85640-3

[CR51] Punt M, van den Brule T, Teertstra WR, Dijksterhuis J, den Besten HMW, Ohm RA, Wösten HAB (2020) Impact of maturation and growth temperature on cell-size distribution, heat-resistance, compatible solute composition and transcription profiles of *Penicillium roqueforti* conidia. Food Res Int 136:109287. 10.1016/J.FOODRES.2020.109287 32846509 10.1016/j.foodres.2020.109287

[CR74] Rambaut A (2012) FigTree version 1.4.0. http://tree.bio.ed.ac.uk/software/figtree/

[CR52] Sant’Ana AS, Rosenthal A, Massaguer PR (2009) Heat resistance and the effects of continuous pasteurization on the inactivation of *Byssochlamys fulva* ascospores in clarified apple juice. J Appl Microbiol 107:197–209. 10.1111/J.1365-2672.2009.04195.X 19298507 10.1111/j.1365-2672.2009.04195.x

[CR53] Santos JLP, Samapundo S, Gülay SM, Van Impe J, Sant’Ana AS, Devlieghere F (2018) Inter- and intra-species variability in heat resistance and the effect of heat treatment intensity on subsequent growth of *Byssochlamys fulva* and *Byssochlamys nivea*. Int J Food Microbiol 279(3):80–87. 10.1016/j.ijfoodmicro.2018.04.035 29751279 10.1016/j.ijfoodmicro.2018.04.035

[CR54] Savi GD, Piacentini KC, Rocha LO, Carnielli-Queiroz L, Furtado BG, Scussel R, Zanoni ET, Machado-de-Ávila RA, Corrêa B, Angioletto E (2018) Incidence of toxigenic fungi and zearalenone in rice grains from Brazil. Int J Food Microbiol 270:5–13. 10.1016/j.ijfoodmicro.2018.02.004 29428818 10.1016/j.ijfoodmicro.2018.02.004

[CR55] Seekles SJ, Teunisse PPP, Punt M, van den Brule T, Dijksterhuis J, Houbraken J, Wösten HAB, Ram AFJ (2021) Preservation stress resistance of melanin deficient conidia from Paecilomyces variotii and Penicillium roqueforti mutants generated via CRISPR/Cas9 genome editing. Fungal Biol Biotechnol 8:4. 10.1186/S40694-021-00111-W 33795004 10.1186/s40694-021-00111-w PMC8017634

[CR56] Snyder AB (2022) The role of heat resistance in yeast spoilage of thermally processed foods: highlighting the need for a probabilistic, systems-based approach to microbial quality. Curr Opin Food Sci 46:100852. 10.1016/J.COFS.2022.100852

[CR57] Souza PBA, Poltronieri KF, Alvarenga VO, Granato D, Rodriguez ADD, Sant’Ana AS, Peña WEL (2017) Modeling of *Byssochamys nivea* and *Neosartorya fischeri* inactivation in papaya and pineapple juices as a function of temperature and soluble solids content. LWT. 10.1016/j.lwt.2017.04.021

[CR58] Stefanello A, Magrini LN, Lemos JG, Garcia MV, Bernardi AO, Cichoski AJ, Copetti MV (2020) Comparison of electrolized water and multiple chemical sanitizer action against heat-resistant molds (HRM). Int J Food Microbiol 335:108856. 10.1016/J.IJFOODMICRO.2020.108856 32961522 10.1016/j.ijfoodmicro.2020.108856

[CR59] Steiner B, Aquino VR, Paz AA, Silla L, Zavascki A, Goldani LZ (2013) *Paecilomyces variotii* as an emergent pathogenic agent of pneumonia. Case Rep Infect Dis. 10.1155/2013/273848 23819077 10.1155/2013/273848 PMC3683431

[CR73] Swofford DL (2002) PAUP Phylogenetic analysis using parsimony (and othermethods) version 4.0b10. https://phylosolutions.com/paup-test/

[CR60] Tong C, Luo J, Xie C, Wei J, Pan G, Zhou Z, Li C (2023) Characterization and biological activities of melanin from the medicinal fungi *Ophiocordyceps sinensis*. Int J Mol Sci. 10.3390/ijms241210282 37373428 10.3390/ijms241210282 PMC10299297

[CR61] Tralamazza SM, Piacentini KC, Savi GD, Carnielli-Queiroz L, de Carvalho Fontes L, Martins CS, Corrêa B, Rocha LO (2021) Wild rice (*O. latifolia*) from natural ecosystems in the Pantanal region of Brazil: host to *Fusarium incarnatum-equiseti* species complex and highly contaminated by zearalenone. Int J Food Microbiol. 10.1016/j.ijfoodmicro.2021.109127 33689972 10.1016/j.ijfoodmicro.2021.109127

[CR62] Tuomela M, Vikman M, Hatakka A, Itävaara M (2000) Biodegradation of lignin in a compost environment: a review. Bioresour Technol 72(2):169–183. 10.1016/S0960-8524(99)00104-2

[CR63] Ulusoy BH, Hamed NS, Yıldırım FK (2022) Heat-resistant moulds: Assessment, prevention and their consequences for food safety and public health. Czech J Food Sci. 10.17221/26/2022-CJFS

[CR64] Urquhart AS, Idnurm A (2023) A polyphasic approach including whole genome sequencing reveals *Paecilomyces paravariotii* sp. nov. as a cryptic sister species to *P. variotii*. J Fungi. 10.3390/jof9030285 10.3390/jof9030285 PMC10055108 36983453

[CR65] van den Brule T, Lee CLS, Houbraken J, Haas PJ, Wösten H, Dijksterhuis J (2020a) Conidial heat resistance of various strains of the food spoilage fungus Paecilomyces variotii correlates with mean spore size, spore shape and size distribution. Food Res Int 137:109514. 10.1016/J.FOODRES.2020.109514 33233149 10.1016/j.foodres.2020.109514

[CR43] van den Brule T, Punt M, Seekles SJ, Segers FJJ, Houbraken J, Hazeleger WC, Ram AFJ, Wösten HAB, Zwietering MH, Dijksterhuis J, den Besten HMW (2022) Intraspecific variability in heat resistance of fungal conidia. Food Res Int 156:111302. 10.1016/J.FOODRES.2022.111302 35651062 10.1016/j.foodres.2022.111302

[CR66] van den Brule T, Punt M, Teertstra W, Houbraken J, Wösten H, Dijksterhuis J (2020b) The most heat-resistant conidia observed to date are formed by distinct strains of Paecilomyces variotii. Environ Microbiol 22:986–999. 10.1111/1462-2920.14791 31444981 10.1111/1462-2920.14791 PMC7065192

[CR67] van den Seekles SJ, Punt M, Dijksterhuis J, Arentshorst M, Ijadpanahsaravi M, Roseboom W, Meuken G, Ongenae V, Zwerus J, Ohm RA, Kramer G, Wösten HAB, de Winde JH, Ram AFJ (2023) Compatible solutes determine the heat resistance of conidia. Fungal Biol Biotechnol 10:1–18. 10.1186/S40694-023-00168-9 37957766 10.1186/s40694-023-00168-9 PMC10644514

[CR68] Visagie CM, Cruywagen EM, Duong TA (2024) A new *Paecilomyces* from wooden utility poles in South Africa. Fungal Syst Evol 13:163–181. 10.3114/fuse.2024.13.10 39140099 10.3114/fuse.2024.13.10 PMC11319801

[CR69] Visagie CM, Houbraken J, Overy DP, Sklenáˇ F, Bensch K, Frisvad JC, Mack J, Perrone G, Samson RA, van Vuuren NI, Yilmaz N, Hubka V (2025) From chaos to tranquillity: a modern approach to the identification, nomenclature and phylogeny of *Aspergillus Penicillium* and other Eurotiales updated accepted species list. Stud Mycol 112:117–260. 10.3114/SIM.2025.112.04 41522877 10.3114/sim.2025.112.04 PMC12786731

[CR70] Vogt G, Vogt G (2022) Environmental adaptation of genetically uniform organisms with the help of epigenetic mechanisms—an insightful perspective on ecoepigenetics. Epigenomes 7:7. 10.3390/EPIGENOMES7010001 10.3390/epigenomes7010001 PMC9844400 36648862

[CR71] Xiao W, Zhang J, Huang J, Xin C, Li MJ, Song Z (2022) Response and regulatory mechanisms of heat resistance in pathogenic fungi. Appl Microbiol Biotechnol 106:5415. 10.1007/S00253-022-12119-2 35941254 10.1007/s00253-022-12119-2 PMC9360699

[CR72] Zhang S, Zhang L, Lan R, Zhou X, Kou X, Wang S (2018) Thermal inactivation of *Aspergillus flavus* in peanut kernels as influenced by temperature, water activity and heating rate. Food Microbiol 76:237–244. 10.1016/J.FM.2018.05.015 30166147 10.1016/j.fm.2018.05.015

