# Supplementary figures and images for: Thermal and oxidative stress responses of Paecilomyces species recovered from beverage processing environments
Source: World J Microbiol Biotechnol. 2026 Apr 27;42(5):239. doi: 10.1007/s11274-026-04970-6 (PMC13111517; doi:10.1007/s11274-026-04970-6)

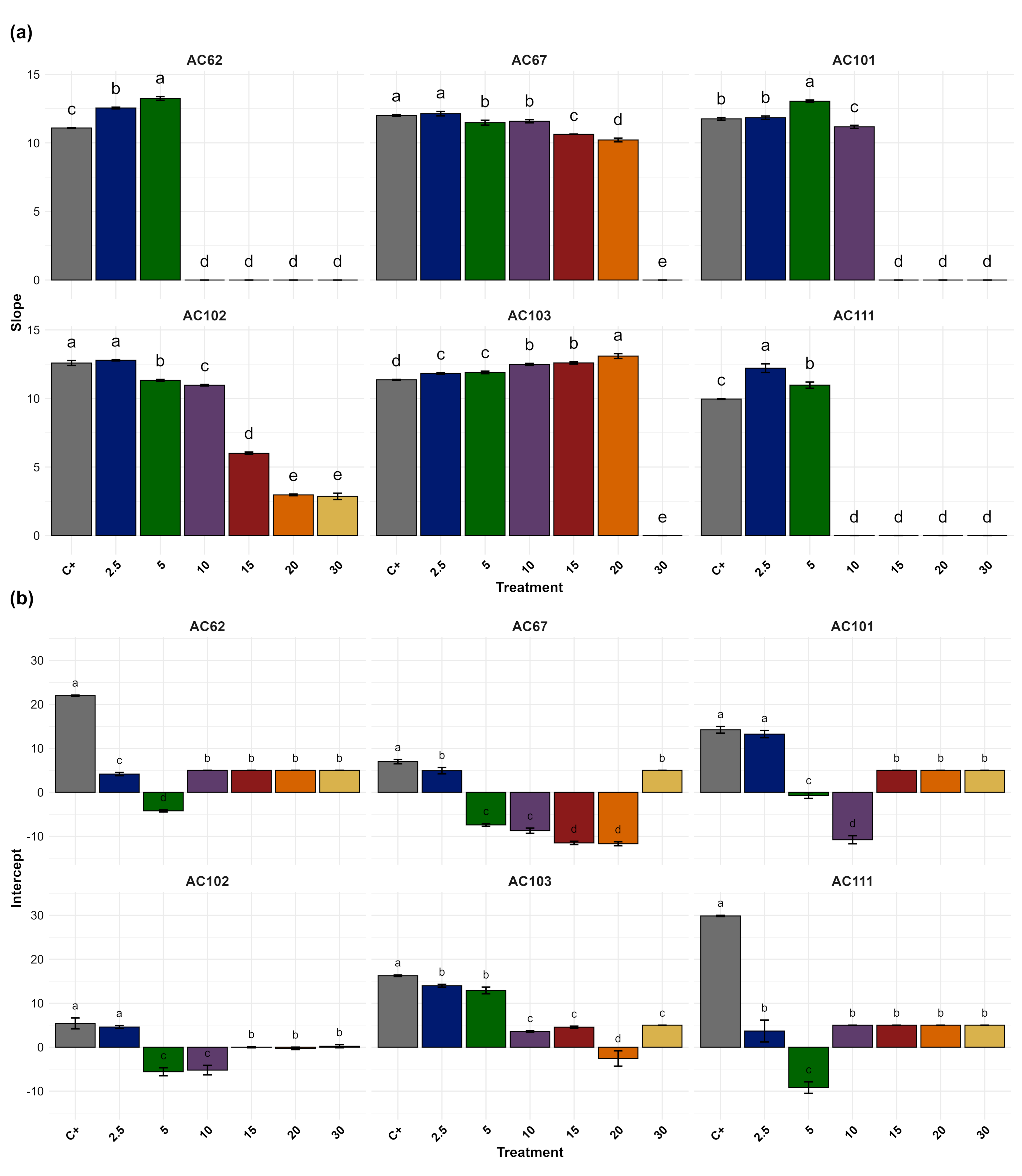

Supplement: Supplementary file 1 — (PNG 912 KB) [file 11274_2026_4970_MOESM1_ESM.png]
