# Supplementary material for: Thermal and oxidative stress responses of Paecilomyces species recovered from beverage processing environments
Source: World J Microbiol Biotechnol. 2026 Apr 27;42(5):239. doi: 10.1007/s11274-026-04970-6 (PMC13111517; doi:10.1007/s11274-026-04970-6)
Supplement: Supplementary file 2 — (DOCX 20.2 KB) [file 11274_2026_4970_MOESM2_ESM.docx]

**Fig. S1.** Intercept **(a)** and slope **(b)** values of six *Paecilomyces* strains (AC 62, AC 67, AC 101, AC 102, AC 103, and AC 111) exposed to different gliding arc plasma jet treatment times (2.5, 5, 10, 15, 20, and 30 min) and the positive control (C, no plasma treatment). Slope and intercept were obtained by linear regression using the complete radial growth data from 0 to 8 days, allowing standardized comparison among strains and treatments. Bars represent mean ± standard error (n = 3). Distinct letters indicate significant statistical differences (p < 0.05).
